# Safety of pregnancy in acromegaly patients and maternal and infant outcomes after pregnancy: single-center experience from China and review of the literature

**DOI:** 10.1186/s12902-023-01341-2

**Published:** 2023-05-09

**Authors:** Rui Jiao, Jianghua Ju, Linjie Wang, Hongbo Yang, Yong Yao, Kan Deng, Huijuan Zhu, Lian Duan

**Affiliations:** 1 Key Laboratory of Endocrinology of National Health Commission, Department of Endocrinology, State Key Laboratory of Complex Severe and Rare Diseases, Peking Union Medical College Hospital, Chinese Academy of Medical Science and Peking Union Medical College, No.1 Shuaifuyuan, Dongcheng District, Beijing, 100730 China; 2 Department of Endocrinology, Qilu Hospital (Qingdao), Cheeloo College of Medicine, Shandong University, Qingdao, Shandong China; 3 Department of Neurosurgery, Peking Union Medical College Hospital, Chinese Academy of Medical Science and Peking Union Medical College, Beijing, China

**Keywords:** Acromegaly, Pregnancy, Fetal malformation, Maternal outcomes

## Abstract

**Background:**

Pregnancy in acromegaly is uncommon and still in debate for fear of tumor progression or potential threat to both mother and fetus’s health. Besides, the data for pregnancy complications in uncontrolled acromegaly is limited. Thus, the objective of this study was to summarize pregnancy safety and disease courses after pregnancy in acromegalic patients and review their clinical characteristics based on disease activity in the literature.

**Methods:**

An evaluation of eight acromegalic women from Peking Union Medical College Hospital (PUMCH) with 11 pregnancies was conducted. We also summarized a literature review of 82 disease-active pregnancies and 63 disease-controlled pregnancies with acromegaly. A second analysis was conducted to compare pregnancy courses and outcomes in different disease activities.

**Results:**

Before pregnancy, all patients had macroadenomas and underwent pituitary surgery. Pregnancy occurred at a median of 6 years (4–10) after the diagnosis of acromegaly. Assisted reproductive therapy was needed in 42.9% of participants. No cases had a premature birth or congenital malformations. Biochemical control was achieved in 50% of females before pregnancy and 75% at the last follow-up after delivery. Data analysis showed no differences in the prevalence of gestational diabetes mellitus (GDM) or pregnancy-induced hypertension (PIH) between acromegaly-active or acromegaly-controlled groups. The GDM prevalence in patients diagnosed during pregnancy (33.3%) was higher than that in patients diagnosed before pregnancy (4.8%) (*p =* 0.001).

**Conclusion:**

Pregnancy without biochemical control in acromegaly and receiving medical treatment during pregnancy are not rare and generally safe for the fetus. There could be a higher prevalence of PIH in acromegalic pregnancies. The treatment of acromegaly and related complications can be managed with regular follow-up after pregnancy.

**Supplementary Information:**

The online version contains supplementary material available at 10.1186/s12902-023-01341-2.

## Background

Acromegaly is almost caused by growth hormone (GH)-secreting pituitary adenoma and is accompanied by an increase in GH and insulin-like growth factor 1 (IGF-1). It significantly contributes to impaired fertility. Acromegalic women of bearing age may have irregular menstruation or even amenorrhea due to gonadotropin deficiency, direct tumor compression and/or hyperprolactinemia [1, 2]. According to previous studies, 30.4–66% of acromegalic women have irregular menses [3, 4]. Symptoms and comorbidities of acromegaly may be alleviated with treatment. In the Chinese acromegalic cohort, the percentage of menstrual disorders in controlled female patients (38.1%) was much lower than that of uncontrolled patients (53.1%) (p < 0.0001) [4]. The development of assisted reproductive therapy (ART) has been gradually applied to pregnancy in acromegalic women of reproductive age [5].

Although pregnancy in acromegaly was uncommon in the past for fear of tumor progression or potential threat to both mother and fetus’s health, more reports suggest that acromegalic pregnancy was generally safe for controlled patients [5, 6].

However, the prevalence of pregnancy complications in acromegalic women is still debatable. As a result of autonomous secretion from the adenoma, the negative feedback effect of IGF-1 on pituitary GH (pGH) levels fails to work. Combined with placental variant GH (vGH) production, pregnant women with acromegaly have higher than normal women [7, 8]. The risks of pregnancy complications, including gestational diabetes mellitus (GDM), pregnancy-induced hypertension (PIH), and adverse fetal consequences, were assumed to be higher in pregnant women with acromegaly due to the changes of the GH-IGF-1 axis. Vialon et al. [9] did a literature review about gestational diabetes and acromegaly, which showed that rare cases of GDM with acromegaly were reported (only 5 GDM in 147 pregnancies) by the end of 2019 [7, 10]. Simultaneously, Vialon et al. [9] showed a 50% prevalence of GDM in a cohort of women with acromegaly in their study, which was much higher than that of the earlier studies. Thus, the data for women in active acromegaly are still limited, and few studies about acromegalic pregnancy have been conducted in China or even in Asia. Furthermore, few studies have been conducted to summarize the long-term course of acromegalic patients after pregnancy. Therefore, this study aimed to summarize and analyze the data from Chinese acromegalic patients with pregnancies who received treatment in PUMCH, particularly the acromegaly activity after pregnancy. Moreover, the study also reviewed the pregnancy process reported in the previous literature according to disease activity to summarize the pregnancy safety of acromegaly patients in different biochemical states.

## Methods

### Patients

Eight patients diagnosed with acromegaly before pregnancy received treatment in PUMCH between January 2004 and January 2021. The inclusion criteria were: (1) confirmed diagnosis of acromegaly before pregnancy or during the first trimester of pregnancy; (2) confirmed pituitary tumor determined by sellar magnetic resonance imaging (MRI) scan. The exclusion criteria were: (1) acromegaly diagnosed after the first trimester of pregnancy; (2) no follow-up after pregnancy. Written informed consent was obtained from all these patients, and the ethics committee approved the study of PUMCH.

### Diagnostic criteria

The diagnosis of acromegaly was based on imaging examination with confirmed pituitary tumor in MRI scan and laboratory examinations of elevated IGF-1 levels and inability to suppress plasma GH (< 1.0 ng/mL) after glucose GH inhibition tests [11]. The definition of disease control was determined based on the updated key points of the Chinese consensus (2021 edition) for the diagnosis and treatment of acromegaly [12]: random serum GH or nadir GH with oral glucose tolerance test (OGTT) < 1.0 µg/L or IGF-1 levels within the normal range for age-and sex-matched healthy individuals. Infants with newborn weight less than 2499 g were referred to as low birth weight regardless of gestational age. Neonatal macrosomia was referred to as a birth weight larger than 4000 g. According to the ICD-10, pregnancy loss includes spontaneous abortion or miscarriage, therapeutic abortion, and fetal death [13]. Premature birth is referred to as live birth before 37 weeks of gestation [13].

### Clinical data collection

We collected demographic and clinical data at baseline, including age, menses, and previous medical and reproductive history. We obtained a sellar MRI and performed laboratory examinations for GH and IGF-1. Information on acromegaly treatments, comprising surgery, radiotherapy, and medical therapy, such as octreotide, lanreotide, pasireotide, and bromocriptine, was also collected. GH and IGF-1 were measured using the fully automatic, solid-phase, and two-site, chemiluminescent enzyme immunometric assay (Immulite 2000, Siemens Healthcare Diagnostics, GH calibrated against the recommended IS 98/574). Direct chemiluminescence immunoassay (Siemens ADVIA Centaur) was used to measure serum and urine cortisol. ACTH samples were delivered to the laboratory on ice and measured by chemiluminescence immunoassay (Siemens IMMULITE 2000). Patient-reported symptoms and signs, such as headaches or visual problems associated with acromegaly, were recorded at diagnosis and pregnancy following up.

### Systematic literature review

We performed a literature search using the PubMed search engine to assess prior reports about pregnant women with acromegaly by December 30, 2022, and data were reviewed. The keyword combinations were as follows: “acromegaly” and “pregnancy”, “acromegaly and pregnancy”.

In this literature review, pregnant women with acromegaly in previous studies were included regardless of whether they had received treatment. Patients were excluded if their clinical data were not available in the literature or their disease condition was not definite before pregnancy. Single case reports were excluded from being able to study the impact of disease control on pregnancy outcomes.

### Statistical analysis

For categorical variables, demographic and descriptive data were presented as numbers (%), and continuous variables were represented as the mean (SD) or median (IQR). The Shapiro-Wilk test was used to test the normality distribution of data. Student’s t-test or the Mann Whitney U test was used to compare continuous variables, and the chi-squared test (or Fisher’s exact test, if required) was used for categorical variables between subgroups. All the conducted tests were two-tailed with a significance level of 0.05. SPSS 25.0 was used for statistical analyses.

## Results

In our cohort, eight acromegalic patients with 11 pregnancies were included. One patient delivered twins. All patients had macroadenomas with maximal tumor diameters ranging from 2.2 to 3.8 cm (2.88 ± 0.70). Patients with acromegaly ranged in age from 16 to 28 years (22.8 ± 4.3). Random GH levels at diagnosis ranged from 6.0 µg/L to 50.0 µg/L, and the percentage of the upper limit of the normal range of IGF-1 ranged from 1.06 to 3.38. Three of the eight patients exhibited menstrual disorders: 1 patient (12.5%) with irregular menses and 2 patients (25%) with amenorrhea. Patient 3 experienced arrested fetal development at 38w when diagnosed with acromegaly; therefore, only her second pregnancy was evaluated in this study. The other patients got their first pregnancy after the diagnosis of acromegaly. The clinical and demographic characteristics of the eight patients are shown in Table 1.

**Table 1:** Clinical characteristics of the eight patients at acromegaly diagnosis

Patient	Age	Maximal tumor diameter (cm)	GH (µg/L)	IGF-1 (ng/ml)	IGF-1 (ULN)	Menses
1	27	NA	50.0	NA	NA	Normal
2	19	3.8	39.3	536	1.65	Amenorrhoea
3	28	NA	3.39	572	2.39	Arrested fetal development at 38w
4	21	3.0	NA	NA	NA	Normal
5	21	2.2	11.7	708	2.55	Irregular
6	16	NA	6.0	404	1.06	Amenorrhoea
7	23	NA	36.2	937	3.38	Normal
8	27	2.5	36.3	460	1.92	Normal

ULN, percentage of the upper limit of normal range of IGF-1; NA, not available.

Prior to pregnancy, all patients underwent surgery. Besides, three (37.5%) patients underwent gamma knife radiosurgery (GKRS), while two (25%) patients received single-fraction radiotherapy (SFRT). Four (50%) patients had combined with hypopituitarism. Adjuvant pharmacotherapy, including octreotide, lanreotide, pasireotide, and bromocriptine, was exerted before 63.3% of pregnancies. Four (50%) patients achieved acromegaly control before pregnancy. Prior to pregnancy, patient 8 had diabetes mellitus, and patient 7 had hypertension. The remaining six patients were free from either of the two complications. Table 2 summarizes the clinical characteristics and treatment modalities of patients with acromegaly before conception.

**Table 2:** Treatment and modalities before conception in patients with acromegaly

No	Surgery	Radiotherapy	Medication	Pituitary trophic hormone deficiency	Acromegaly control	Hypertension	DM
1a	Yes	GKRS	Oct Lan	GTD ACTH, TSH	Yes	No	No
1b	Yes	GKRS	No	GTD ACTH, TSH	Yes	No	No
2a	Yes	No	Oct	No	No	No	No
2b	Yes	No	Oct	No	No	No	No
3	Yes	No	Br	No`	No	No	No
4a	Yes	GKRS	Br Pas	No	No	No	No
4b	Yes	GKRS	No	No	Yes	No	No
5	Yes	SFRT	Oct	GTD TSH	Yes	No	No
6	Yes	SFRT	No	GTD	Yes		
7	Yes	GKRS	Oct	No	No	Yes	No
8	Yes	No	No	TSH	No	No	Yes

GKRS, gamma knife radiosurgery; SFRT, single-fraction radiotherapy; Oct, octreotide; Lan, lanreotide; Br, bromocriptine; Pas, pasireotide; GTD, gonadotropin deficiency; TSH def, thyroid stimulating hormone deficiency; ACTH def, adrenocorticotropic hormone deficiency.

Pregnancy occurred at a median of 6 years (4–10) after the diagnosis of acromegaly. Three (42.9%) patients received ART, including one with in vitro fertilization and embryo transfer (IVF-ET) and two with ovulation induction (OI), all of whom had successful term pregnancies. Abortion occurred in 18.2% of pregnancies. Patient 2 decided to have an abortion during her second pregnancy because of active acromegaly and intense headaches. The first pregnancy of patient 4 was accidental during a clinical drug trial, and she later underwent an induced abortion. All these patients had no visual field defect during pregnancy. Patient 8 had PIH, and patient 5 had GDM. Patient 3 received bromocriptine therapy for acromegalic control during the whole pregnancy. Cesarean section was exerted in five cases. Only one patient (patient 8) experienced premature rupture of fetal membranes. Experienced neonatologists examined all infants, and they were found to have no congenital anomalies among them. The pregnancy and infant outcomes of patients with acromegaly are represented in Table 3.

**Table 3:** Pregnancy and infant outcomes in patients with acromegaly

No	Age	ART	PIH	GDM	Medical therapy at pregnancy	Pregnancy Loss (w)	Delivery	Gestation	Newborn weight (g)	Breastfeeding
1a	37	IVF-ET	No	No	No	No	C/S	NA	3150	NA
1b	42	IVF-ET	No	No	No	No	C/S	38 + 3	2860	NA
2a	24	No	No	No	No	No	SVD	40	NA	NA
2b	26	No	No	No	No	Yes^c^	Non	Non	Non	Non
3	31	No	No	No	Br	No	C/S	37 + 3	3450	Yes-2 mo
4a	24	No	No	No	No	12	Non	Non	Non	Non
4b	25	No	No	No	No	No	SVD	40 + 1	2900	Yes-2 mo
5	27	OI	No	Yes	No	No	C/S	38	NA	NA
6	29	OI	No	No	No	No	SVD	40	3200	Yes-14 mo
7	30	NA	No^a^	No	No	No	C/S	40	NA	NA
8	31	No	Yes (37w)	No^b^	No	No	SVD	38 + 6	3250	NA

ART, assisted reproduction therapy; IVF-ET, in vitro fertilization and embryo transfer; OI, ovulation induction; PIH, pregnancy induced hypertension; GDM, gestational diabetes mellitus; Br, Bromocriptine; C/S, caesarean section; SVD, spontaneous vaginal delivery; NA, not available.

^a^: Hypertension diagnosed before pregnancy

^b^: Diabetes mellitus diagnosed before pregnancy

^c^: Induced abortion because of active acromegaly with intense headache.

After delivery, the median follow-up time was 29 months (range, 3–93 months) in the cohort. Totally six patients achieved biochemical control at the last follow-up. Before pregnancy, all the patients who had controlled acromegaly remained stable. Patient 2 and patient 3 without biochemical control before pregnancy were pulled off by multidisciplinary treatment after delivery, both of whom underwent surgery, SFRT, and medical treatment after delivery. About half of the patients in the cohort received continuous acromegalic treatment after pregnancy. A second transsphenoidal pituitary adenoma resection was done in patient 2 and patient 3 after delivery. Three patients underwent radiotherapy, two (patient 2 and patient 3) received SFRT, and one (patient 7) received a cyber-knife. Four patients (2, 3, 4, 7) received medical therapy. In terms of the complication of acromegaly at the last follow-up, patient 5 returned to normal blood glucose levels after delivery, but patient 8 still had hypertension. Two patients had hypertension, and one had diabetes mellitus. Table 4. shows the disease condition and treatment after delivery in patients with acromegaly and their clinical characteristics at the last follow-up.

**Table 4:** Disease condition and treatment after delivery in patients with acromegaly and clinical characteristics at last follow-up

No	Before pregnancy		Follow up after last pregnancy (months)	Biochemical examination at last follow-up			Acromegalic treatment after pregnancy			Clinical characteristics at last follow-up		
	Acromegaly control	Medical therapy		GH (µg/L)	IGF-1 (ng/ml)	IGF-1 (ULN)	Surgery	Radiotherapy	Medical therapy	Acromegaly control	HTN	DM
1	Yes	Oct Lan	32	0.7	191	0.71	No	No	No	Yes	No	No
2	No	Oct	86	0.639^a^	111.6	0.36	Yes	SFRT	Oct	Yes	No	No
3	No	No	93	0.9	216	0.76	Yes	SFRT	Br Oct	Yes	No	No
4	Yes	Br Pas	35	3.4	289	0.87	No	No	Br Cab	Yes	No	No
5	Yes	Oct	3	0.3	303	0.92	No	No	No	Yes	No	No
6	Yes	No	14	0.932^a^	441	1.34	No	No	No	Yes	No	No
7	No	Oct	18	3.9	471	1.53	No	Cyber knife	Oct	No	Yes	No
8	No	No	26	4.01^a^	845	2.78	No	No	No	No	Yes	Yes

ULN, percentage of the upper limit of normal range of IGF-1; SFRT, single-fraction radiotherapy; Oct, Octreotide; Br, Bromocriptine; Cab, Cabergoline; HTN, hypertension; DM, diabetes mellitus.

^a^: Nadir GH with OGTT.

**Fig. 1:**
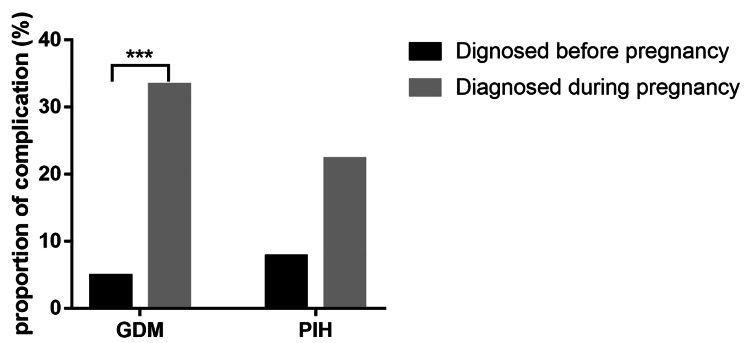
Proportion of GDM and PIH in patients with diagnosis during pregnancy or before pregnancy in literature review

A review of the literature [5–7, 10, 14–16] reveals that pregnancy in disease-active women with acromegaly was not rare. 8 studies involving 145 pragnancies met inclusion criteria (Supplementary Tables 1 and Supplementary Fig. 1) [34]. Eighty-two disease-active cases (56.55%) in 145 pregnancies failed to control GH/IGF-1 hypersecretion before pregnancy (Table 5). The average age of women at the pregnancy diagnosis is 31.4 ± 4.7 years old, 91.8% of whom had GH-secreting pituitary macroadenoma. 90.3% of patients underwent pituitary surgery, 28.2% had radiotherapy, and 71.7% had medical treatment before pregnancy. Nine patients were diagnosed during pregnancy, all of whom were in active disease. As shown in Fig. 1, the proportion of GDM in patients diagnosed during pregnancy (33.3%) was higher than that in patients diagnosed before pregnancy (4.8%) (*p* = 0.001). There was no significant difference between the disease-active and the disease-controlled groups in terms of average age, tumor size, abortion, the use of ART, or the number of patients with a history of radiotherapy or medical treatment before pregnancy. However, more women were medically treated before pregnancy in the disease-controlled group than in the disease-active group (84.51% vs. 96.83%; *p* = 0.016). There were no statistical differences between the two groups in terms of the prevalence of GDM, PIH, headaches, or delivery modes. The prevalence of premature birth for patients with active disease conditions was slightly higher than that of patients with disease control, though the difference was not statistically significant (16.67% versus 2.63%; *p* = 0.114). The newborn weight presented no difference between the two subgroups. On the other hand, compared with patients achieving disease control, patients with active acromegaly tended to have a higher proportion of microsome (7.69% vs. 0%; *p* = 0.069) and macrosome (7.69% vs. 0%; *p* = 0.069), though the difference was not statistically significant.

**Fig. 2:**
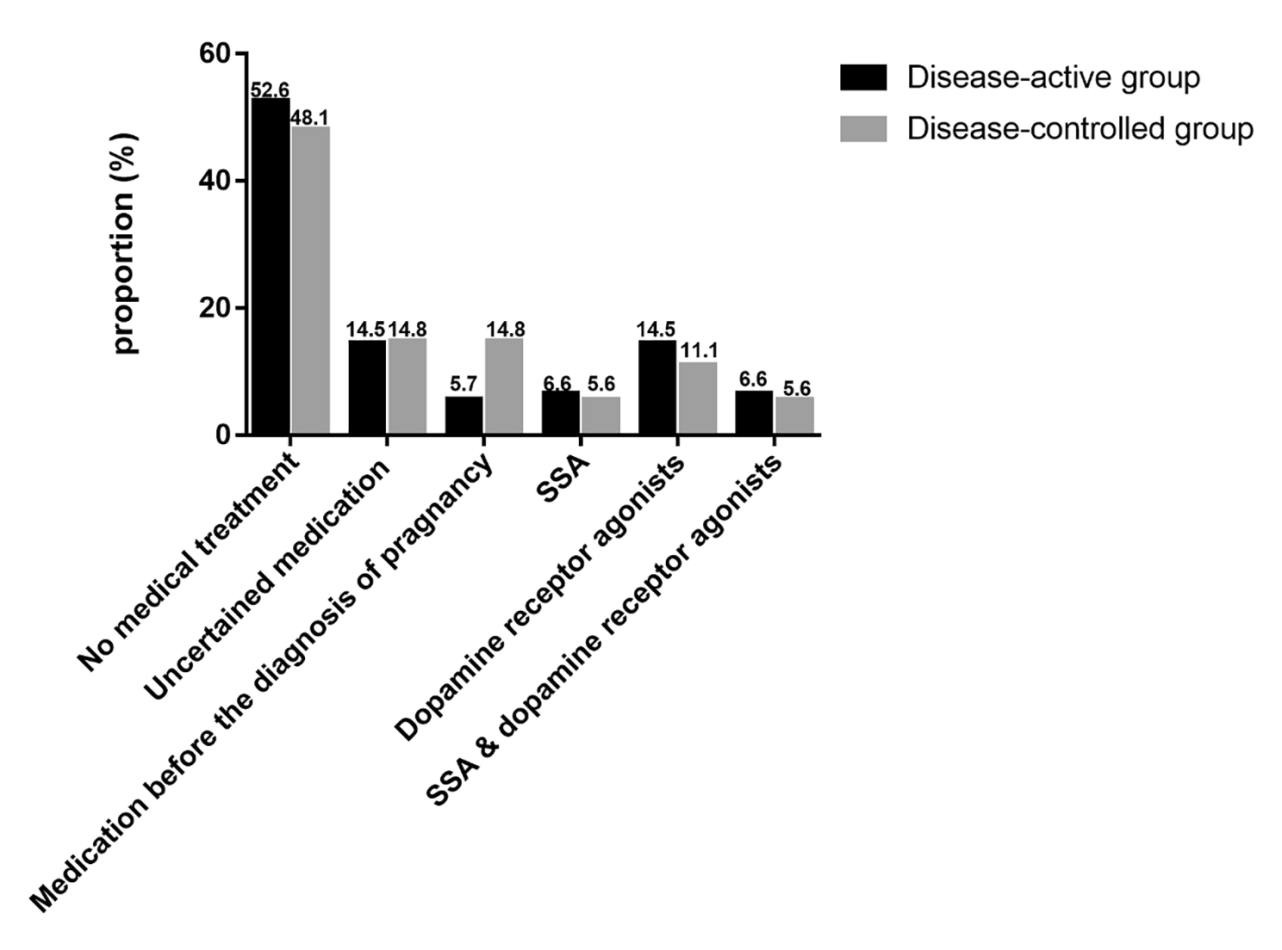
Medication for patients with acromegaly during pregnancy between the disease-controlled and disease-active groups in the literature review

Figure 2 shows medication for patients with acromegaly during pregnancy. Totally 12 (9.2%) patients discontinued medication for acromegaly after pregnancy diagnosis, with 5.7%in the disease-active group and 14.8%in the disease-controlled group. Medical therapy during pregnancy was used in 22.2% and 28.6% of the disease-controlled and disease-active groups, respectively. The proportion of using SSA, dopamine receptor agonists, and the combination of both was 6.6%, 14.5%, 6.6% in the disease-active group, and 5.6%, 11.1%, and 5.6% in the disease-controlled group, respectively.

**Table 5:** Acromegaly patients’ characteristics and pregnancy outcomes during gestation

characteristics	Total N = 145	Disease-active women N = 82			Disease-controlled women N = 63			*p* value
				MD			MD	
Age at the diagnosis of pregnancy				47			27	0.793
Average (SD)	31.4 ± 4.7	31.6 ± 4.9			31.3 ± 4.5			
Min-max	21–43	21–40			24–43			
Diagnosis during pregnancy	9 (8.0)	9	14.06%	18	0	0%	14	0.006
Abortion	6 (4.1)	4	4.88%	0	2	3.17%	0	0.61
ART	6 (20.0)	1	11.1%	73	5	23.81%	42	0.462
Macroadenoma at the diagnosis of acromegaly	123 (91.8)	68	93.15%	9	55	90.16%	2	0.531
Previous treatment								
Surgery	121 (90.3)	60	84.51%	11	61	96.83%	0	0.016
Radiotherapy	29 (28.2)	17	27.42%	20	12	29.27%	22	0.838
Medical treatment	104 (71.7)	56	68.29%	0	48	76.19%	0	0.295
Medical treatment during pregnancy	64 (49.6)	36	48.00%	7	28	51.86%	9	0.666
Gestational diabetes mellitus				0			3	0.981
Yes	10 (7.0)	6	7.32%		4	6.67%		
No	123 (86.6)	71	86.59%		52	86.67%		
T2D before pregnancy	9 (6.3)	5	6.09%		4	6.67%		
Gestational hypertension				0			1	0.153
Yes	16 (11.1)	13	15.85%		3	4.84%		
No	117 (81.3)	63	76.83%		54	87.10%		
Eclampsia	1 (0.7)	1	1.22%		0	0		
Hypertension before pregnancy	10 (6.9)	5	6.09%		5	8.06%		
Headaches	30 (27.5)	21	33.33%	19	9	19.57%	17	0.112
Delivery				52			34	0.460
Vaginal	24 (40.0)	11	35.48%		13	44.83%		
Cesarean	36 (60.0)	20	64.52%		16	55.17%		
Premature birth	7 (9.5)	6	16.67%	46	1	2.63%	25	0.114
Gemellary pregnancy		1	2.78%		0			
Singleton pregnancy		5	13.89%		1	2.63%		
Weight of newborns (kg)				44			38	0.57
Average (SD)	3.26 ± 0.55	3.23 ± 0.63			3.31 ± 0.40			
Min-max	1.30–4.30	1.30–4.30			2.59–4.26			
Weight of newborns				17			22	
Microsome	5 (4.7)	5	7.69%		0	0		0.069
Macrosome	5 (4.7)	5	7.69%		0	0		0.069

N, number of patients; MD, missing data; GDM, gestational diabetes mellitus, T2D, type-2 diabetes mellitus

## Discussion

Based on a cohort of pregnant women with acromegaly, this study found that pregnancy in acromegaly is generally safe, but it is essential to catch with the right timing for a lower risk of tumor impression and complications.

The present study showed the outcomes of 11 cases of acromegalic pregnancy and the long-term acromegaly courses of eight patients after pregnancy. Acromegaly can have an insidious onset, delaying diagnosis as long as 4.5–9 years. Patients with acromegaly are typically in their fifth decade at diagnosis (19). Given this perspective, some researchers used to think reducing pregnancy may be a sensible choice considering the risk of tumor progression and pregnancy complications for elderly acromegalic women of childbearing age [17, 18]. However, more acromegalic patients can be diagnosed early with a deeper physician’s awareness of the disease [19, 20]. The mean age of acromegaly diagnosis in the current cohort was 22.8 years old, while the mean pregnancy age was 29.6 years old. It was essential for these patients of reproductive age to adequately account for their fertility willingness and acromegalic condition.

Impaired fertility can be caused not only by the disease impact of acromegaly but also by hypopituitarism due to surgery and radiotherapy [21]. In China, the proportion of menstrual disturbance was about 60% in females with acromegaly at diagnosis [4, 22]. Even in patients undergoing treatment, the proportion was still up to 40% [4]. With the development of assisted reproductive technology, such as OI, intrauterine insemination, and in vitro fertilization, there have been studies reporting the use of ART in acromegalic women with a proportion of about 20% [5]. A quarter of the cohort received ovulation induction in this study, and 12.5% of patients received IVF-ET.

Although the guideline opposed monitoring GH and/or IGF-1 levels during pregnancy (26), a potential threat for acromegaly progression still exists. There was an 18.2% rate of abortions, one of which was severe headaches during the first trimester, which suggested that clinical signs and symptoms associated with acromegaly can implicate essential imaging screening and are vital to avoid the neglect of disease progression.

To determine pregnancy safety in patients with active and controlled stages of acromegaly, we summarized their characteristics based on reported studies. There have been about 200 pregnancies with acromegaly reported [7, 10, 23]. The prevalence of pregnancy with active acromegaly was more than that of achieving biochemical control, with 82 cases of the former and 63 cases of the latter in this literature review. There were no congenital malformations in either disease-active or disease-controlled groups. Half of the patients failed to get pregestational biochemical control in our cohort but with no neonatal malformation or serious adverse maternal events. The above evidence suggests the low perniciousness of acromegaly activity in fetal safety.

However, concerns about gestational complications, such as GDM and PIH, in acromegaly pregnancy still exist, particularly in patients with active disease. In the current literature review, we found no difference in the proportion of GDM and hypertension between biochemical active and controlled groups. We observed an 11.1% prevalence of PIH in our cohort, which is higher than the rates of 5.8% reported in a recent study [24]. According to our observation, the marked development of hypertension during pregnancy would be considered the effects of excess GH on sodium retention (28). The prevalence of GDM varied from 9.3 to 19.7% in different areas of China, while we observed an 11.1% rate of GDM in our cohort, similar to that of the reported studies [25–28]. According to a recent meta-analysis, it was uncommon for worsening of preexisting diabetes or development of gestational diabetes with an overall frequency of 9%, lower than that estimated in the general population of pregnant women [29]. However, Vialon et al. [9] recently reported a prevalence of up to 50%, with a 66.7% frequency of GDM when IGF-1 secretion was controlled before pregnancy vs. 37.5% in the pregestational uncontrolled group, which suggested besides classical risk factors of GDM, such as age and BMI, lack of GH/IGF-I hypersecretion control may also aggravate the risk of GDM in acromegaly. Following Vialon’s research (8), the proportion of GDM in patients with acromegaly diagnosed during pregnancy in this study was higher than that in patients with acromegaly diagnosed before pregnancy. Considering pregnancy itself is an insulin-resistant state and GH is a potent insulin antagonist, acromegalic women in pregnancy are more likely to develop gestational glucose intolerance or diabetes mellitus [10]. Screening of GDM may be an appropriate choice to be extended to all women with acromegaly even without classical risk factors.

Previous guidelines recommended withholding acromegaly medical therapy during pregnancy and administering it only for tumor progression and headache control [30]. All patients except patient 3 withdrew before preparing for pregnancy in the present cohort. However, in a practical situation, some acromegaly patients did not withdraw until they were found to be pregnant. In the literature review, we found that 9.2% of patients had exposure to acromegalic medicine by the pregnancy diagnosis but discontinued it after that. And more patients chose to discontinue medication at the diagnosis of pregnancy in the disease-controlled group than that in the disease-active group. About 20–30% of acromegaly patients took medication throughout pregnancy to get acromegalic biochemical control. The disease-active group had a higher proportion which suggested medical therapy was not rare in pregnancy with acromegaly. There was no newborn malformation in any of the patients reviewed in the literature, which suggested receiving medical treatment was generally safe for acromegaly during pregnancy, even in early pregnancy, during which drugs were easy to cause fetal malformations.

Two cases of malformations have been reported in acromegaly pregnancy, with prior reports of congenital cataracts, microcephaly, craniosynostosis (on metformin), and ureteral stenosis (on octreotide) [16, 31]. But by now, it is still difficult to determine the impact of medication for acromegaly as a contributing factor to the observed malformations [16].

During pregnancy, a high estrogen level is believed to be advantageous for acromegalic biochemical control, for it can increase hepatic GH resistance by inhibiting the JAK-STAT pathway [32, 33]. Previous studies showed either betterment or stability of both biochemical control and clinical symptoms in acromegalic patients during pregnancy [6, 34]. The European Society of Endocrinology Clinical Practice Guideline on pituitary adenomas recommends reassessing disease activity after pregnancy [35]. For pregestational disease-active patients in the present research, three-quarters of them received integrative treatment. Two patients achieved biochemical control, whereas the other failed. One postpartum disease-active patient discontinued monitoring GH and IGF-1 levels 3 months after her pregnancy. A quarter of patients lost follow-up, which was bad for managing acromegaly and associated complications. It also suggested strengthening communication and health education with patients to ensure regular follow-up.

Our study had some limitations as well. First, besides a limited number of cases for the rarity in acromegalic pregnancy, there was also a lack of clinical data, such as tumor volume and reassessment for pituitary function after delivery. Second, we did not follow up on the development of these newborns. However, the current study is the first to report acromegalic pregnancy data in China and summarize the long-term changes in disease activity after pregnancy, enriching the clinical experience. Additionally, we analyzed the difference in maternal and fetal outcomes between patients with active and controlled acromegaly in previously reported studies. Prospective studies are needed to explore and provide more evidence about the association between acromegaly and pregnancy.

## Conclusion

Pregnancy without biochemical control in acromegaly and receiving medical treatment during pregnancy are not rare events and are generally safe for the fetus. Catching with the right timing was significant for lowering the risk of tumor progression and complications. There could be a higher prevalence of pregnancy-induced hypertension in acromegalic pregnancies. Failure to get biochemical control and delay in acromegaly diagnosis may increase the risk of GDM. Acromegaly may be controlled by treatment after pregnancy, and regular follow-up is essential for managing acromegaly and related complications.

## Electronic supplementary material

Below is the link to the electronic supplementary material.

Supplementary Material 1

## Data Availability

The datasets used and analysed during the current study are available from the corresponding author on reasonable request.

## List of Abbreviations

PUMCH: Peking Union Medical College Hospital
GDM: gestational diabetes mellitus
PIH: pregnancy-induced hypertension
GH: growth hormone
IGF-1: insulin-like growth factor 1
ART: assisted reproductive therapy
pGH: pituitary GH
vGH: variant GH
MRI: magnetic resonance imaging
GKRS: gamma knife radiosurgery
SFRT: single-fraction radiotherapy
Oct: octreotide
Lan: lanreotide
Br: bromocriptine
Pas: pasireotide
GTD: gonadotropin deficiency
TSH def: thyroid stimulating hormone deficiency
ACTH def: adrenocorticotropic hormone deficiency
IVF-ET: in vitro fertilization and embryo transfer
OI: ovulation induction
C/S: caesarean section
SVD: spontaneous vaginal delivery
ULN: percentage of the upper limit of normal range
HTN: hypertension
DM: diabetes mellitus
MD: missing data
T2D: type-2 diabetes mellitus
