# Supplementary material for: Safety of pregnancy in acromegaly patients and maternal and infant outcomes after pregnancy: single-center experience from China and review of the literature
Source: BMC Endocr Disord. 2023 May 9;23:104. doi: 10.1186/s12902-023-01341-2 (PMC10169299; doi:10.1186/s12902-023-01341-2)

Supplementary Table 1: Literature review: pregnancies in acromegalic women

|  | Number of pregnancies | Geographic Domains | Duration of the study |
| --- | --- | --- | --- |
| Das,2021 | 14 | India | 2010-2019 |
| Guarda, 2020 | 4 | America, China | 2014-2019 |
| Hannon, 2019 | 17 | Ireland | 1995-2017 |
| Jallad,2017 | 31 | Brazil, Israel | NA |
| Dias, 2014 | 10 | Brazil | NA |
| Caron, 2010 | 57-2 | France | NA |
| Cozzi, 2006 | 7 | Italy | 2000-2002 |
| Atmaca,2005 | 5-2 | America | 1987-2003 |

Supplementary Figure 1: Flowchart of literature reviwe


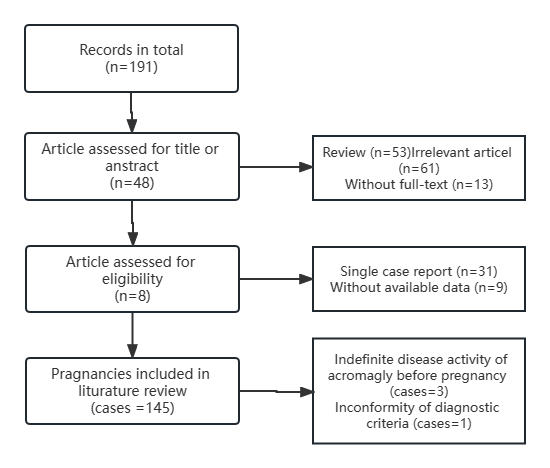

Supplement: Supplementary file 1 — Supplementary Material 1 [file 12902_2023_1341_MOESM1_ESM.docx]
